# Chronische Schmerzen im Seniorenalter vor dem Hintergrund der COVID-19-Pandemie

**DOI:** 10.1007/s00482-022-00663-9

**Published:** 2022-08-26

**Authors:** K. Teichmüller, L. Bast, H. L. Rittner, G. Kindl

**Affiliations:** 1 Zentrum für interdisziplinäre Schmerzmedizin (ZiS), Klinik für Anästhesiologie, Intensivmedizin, Notfallmedizin und Schmerztherapie, Universitätsklinikum Würzburg, Straubmühlweg 2a – Haus A9, 97078 Würzburg, Deutschland; 2 Abteilung Interventionspsychologie, Lehrstuhl für Psychologie I, Institut für Psychologie, Universität Würzburg, Würzburg, Deutschland

**Keywords:** SARS-CoV‑2, Höheres Lebensalter, Biopsychosoziales Schmerzmodell, Deutscher Schmerzfragebogen, Schmerzbedingte Beeinträchtigung, SARS-CoV‑2, Older adults, Biopsychosocial model of pain, German Pain Questionnaire, Pain-related disability

## Abstract

**Hintergrund:**

Internationale Studien belegen negative Auswirkungen der COVID-19-Pandemie auf Stimmung und Stresslevel befragter Personen. Auch konnten Zusammenhänge zwischen der Pandemie und höheren Schmerzstärken sowie stärkerer schmerzbedingter Beeinträchtigung nachgewiesen werden. Die Studienlage dazu, ob ältere Menschen besser oder schlechter mit der Pandemie und ihren Auswirkungen umgehen können als jüngere Personen, ist aber uneindeutig.

**Methodik:**

Seit einigen Jahren bietet das Universitätsklinikum Würzburg ein multimodales Schmerztherapieprogramm für SeniorInnen an. Für die vorliegende Arbeit wurden retrospektiv klinische Routinedaten zum Zeitpunkt des interdisziplinären multimodalen Assessments von *n* = 75 TeilnehmerInnen in den Jahren 2018 und 2019 mit denen von *n* = 42 Patientinnen während der COVID-19-Pandemie 2020–2021 verglichen. Wir untersuchten Schmerz, psychische Belastung und körperliches Funktionsniveau mithilfe des Deutschen Schmerzfragebogens, klinischer Diagnostik und geriatrischer Funktionstests.

**Ergebnisse:**

Die beiden Teilstichproben unterschieden sich nicht in demografischen Merkmalen. Bezüglich Schmerzintensität und Beeinträchtigung sowie der psychischen Belastung fanden sich ebenfalls keine signifikanten Unterschiede. Lediglich die Anzahl der schmerzbedingt beeinträchtigten Tage war vor Corona signifikant höher. Die geriatrischen Funktionstests zeigten signifikant bessere Werte während der Pandemie an.

**Diskussion:**

Die vorliegenden Daten zeigen keine Verschlechterung von Schmerz und körperlichem sowie psychischem Wohlbefinden bei SeniorInnen vor dem Hintergrund der Pandemie. Weitere Studien sollten die möglichen Gründe dafür untersuchen. Diese könnten in einer höheren Resilienz der SeniorInnen basierend auf ihrer Lebenserfahrung, finanziellen Sicherheit oder einer geringeren Veränderung des Lebensalltags liegen.

## Abstract (en)

**Background:**

International studies have shown negative effects of the COVID-19 pandemic on mood and levels of distress. Correlations between the pandemic and higher levels of pain as well as greater pain-related disability have also been found; however, studies report ambiguous results about whether elderly people cope differently with the pandemic and its effects.

**Methods:**

The University Hospital of Würzburg offers multimodal pain therapy for older adults. The current study performed a retrospective analysis of routine data measured during an interdisciplinary multimodal assessment. We compared *n* = 75 patients taking part in the therapy during 2018 and 2019 to *n* = 42 patients assessed in 2020–2021. We measured pain, mental distress and physical functioning using the German Pain Questionnaire, clinical diagnosis, and geriatric tests of physical fitness.

**Results:**

Both subgroups did not differ in demographic characteristics, neither did we find significant differences regarding pain intensity, pain-related disability, and mental health; however, patients before the pandemic reported a higher number of days on which they felt limited due to pain. In the physical performance test, we even found significantly better results during the COVID-19 pandemic.

**Discussion:**

The current data do not support an aggravation of pain or mental and physical well-being. Possible explanations could be better resilience in elderly people due to their experience of life, financial security or less change in their daily life.

Kaum etwas kennzeichnete die vergangenen zwei Jahre so sehr wie die COVID-19-Pandemie. Diese Arbeit beleuchtet die Auswirkungen der Pandemie auf ältere Menschen, die an einer chronischen Schmerzerkrankung leiden, und betrachtet dabei sowohl Schmerzintensität und Beeinträchtigung als auch psychische Belastung und körperliche Funktionalität.

## Einleitung

In Deutschland leben derzeit ca. 18 Mio. Menschen, die über 65 Jahre alt sind [4]. Die Prävalenz chronischer, d. h. seit mind. 3 Monaten anhaltender Schmerzen liegt in dieser Altersgruppe bei 25–76 % [1]. Chronische Schmerzen werden gemäß dem biopsychosozialen Modell durch ein Zusammenspiel aus biologischen, psychologischen und sozialen Einflüssen aufrechterhalten [16] und zeichnen sich durch ein hohes Ausmaß an schmerzbedingter Beeinträchtigung und eine hohe Inanspruchnahme von Gesundheitsleistungen aus [13].

Chronische Schmerzen gehen zudem mit einem erhöhten Risiko für psychische Erkrankungen einher, wobei vor allem Depressionen, Angststörungen und Substanzabusus zu nennen sind [7, 15].

Bezüglich der Prognose psychischer Erkrankungen bei älteren Menschen sind als Risikofaktoren u. a. somatische und Mehrfacherkrankungen, belastende Lebensereignisse und soziale Isolation bekannt [6], wobei v. a. Letzteres vor dem Hintergrund der aktuellen COVID-19-Pandemie besonders zu beachten ist.

Studien aus den USA [11] und Kanada [18] belegen negative Auswirkungen der COVID-19-Pandemie bzw. der Anordnung zur sozialen Distanzierung auf Schmerzstärke und schmerzbedingte Beeinträchtigung der untersuchten Personen. Über 40 % der Befragten äußerten zudem eine erhöhte psychische Belastung. Laut Pagé et al. [18] waren diese Ergebnisse bei älteren Menschen jedoch weniger prävalent als bei jüngeren.

Eine Studie mit deutschen und polnischen Teilnehmenden [2], die jedoch nicht an einer chronischen Schmerzerkrankung litten, kam zu dem Ergebnis, dass ältere Menschen besser mit der Pandemie umzugehen scheinen als jüngere Menschen; sie zeigten beispielsweise weniger Ängstlichkeit und fühlten sich weniger durch das Coronavirus bedroht. Andere Studien fanden allerdings einen genau umgekehrten Zusammenhang [17].

In der vorliegenden Arbeit soll anhand von klinischen Routinedaten aus dem interdisziplinären multimodalen Assessment die Annahme geprüft werden, dass sich die Schmerzsymptomatik, die psychische Belastung sowie das körperliche Funktionsniveau bei älteren Menschen mit chronischer Schmerzerkrankung im Zeitraum der Pandemie verschlechtert haben.

## Methodik

In der Schmerztagesklinik des Zentrums für interdisziplinäre Schmerzmedizin am Universitätsklinikum Würzburg werden in der sogenannten „Seniorengruppe“ ältere Menschen mit chronischen Schmerzerkrankungen behandelt. Dieses multimodale Therapieprogramm umfasst 2 tagesklinische Behandlungstage pro Woche à 6 h über einen Zeitraum von 8 Wochen. Vor Aufnahme werden im Rahmen eines interdisziplinären multimodalen Assessments umfangreiche Tests zur Abklärung von Schmerz- und psychischen Symptomen sowie der körperlichen Leistungsfähigkeit durchgeführt.

### Datenerhebung und Instrumente

Für diese Arbeit wurden retrospektiv die klinischen Routinedaten von Betroffenen, die sich vor Ausrufung der COVID-19-Pandemie (Gruppe „vor Pandemie“: Januar 2018 bis Februar 2020) zur Behandlung vorgestellt haben, mit Daten von Betroffenen, deren Aufnahme während der Pandemie stattfand (Gruppe „während Pandemie“: März 2020 bis Dezember 2021), in einem Between-Design verglichen. Die unabhängige Variable ist somit der Zeitpunkt der Vorstellung mit den Stufen „vor“ bzw. „während der Pandemie“. Das oben erwähnte Therapieprogramm hatte zu diesem Zeitpunkt für die Teilnehmenden noch nicht begonnen. Ein positives Votum der Ethikkommission lag vor. Die ausgewerteten Daten stammen aus dem *Deutschen Schmerzfragebogen* (DSF; [19]) und den Entlassbriefen der behandelten Personen.

Dem *DSF* wurden neben den demografischen Angaben folgende Skalen entnommen:

- Schmerzintensität: momentane, durchschnittliche und größte Schmerzintensität während der letzten 4 Wochen auf einer numerischen Rating-Skala von 0 („kein Schmerz“) bis 10 („stärkster vorstellbarer Schmerz“)
- Schmerzbedingte Beeinträchtigung: Anzahl der schmerzbedingt beeinträchtigten Tage in den letzten 3 Monaten sowie Ausmaß der Einschränkung in Alltag, Freizeit und Arbeitsfähigkeit (numerische Rating-Skala von 0 = „keine Beeinträchtigung“ bis 10 = „völlige Beeinträchtigung“)
- *Marburger Fragebogen zum habituellen Wohlbefinden (MFHW):* Der Fragebogen besteht aus 7 Fragen, die jeweils auf einer Skala von 0 („trifft gar nicht zu“) bis 5 („trifft vollkommen zu“) beantwortet werden. Erfasst wird, ob trotz Schmerzen Wohlbefinden erlebt werden kann, z. B. durch Item 2: „Trotz Schmerzen würde ich sagen: Ich bin innerlich erfüllt gewesen“ oder Item 5: „…: Ich bin mit meiner Arbeitsleistung zufrieden gewesen“. Ein Summenwert von ≤ 10 Punkten bedeutet ein signifikant beeinträchtigtes allgemeines Wohlbefinden.
- *Depressions-Angst-Stress-Skalen (DASS):* Die Auswertung der drei Skalen mit je 7 Items erfolgt durch Bildung des jeweiligen Summenwerts bei 4‑stufigem Antwortformat. Ein Summenwert von ≥ 6 (Angstskala) bzw. ≥ 10 (Depressions- und Stressskala) ist als auffällig zu bewerten.

Anzahl und Art der Schmerz- und psychischen Diagnosen wurden den Entlassbriefen entnommen.

Zur Beurteilung der körperlichen Leistungsfähigkeit wurde die *Short Physical Performance Battery* (*SPPB *[9]) ausgewertet, die im Rahmen der physiotherapeutischen Aufnahme zu Behandlungsbeginn erhoben wird. Die SPPB besteht aus (1) einem Balancetest, bei dem für jeweils 10 s ein Side-by-side-Stand (Füße direkt nebeneinander), ein Semitandemstand (Füße versetzt nebeneinander) und ein Tandemstand (Füße direkt hintereinander) gehalten werden muss, (2) einem Gehgeschwindigkeitstest, in welchem die Zeit für das Zurücklegen einer 4‑Meter-Strecke gemessen wird, und (3) dem Sit-to-stand-Test. Bei Letzterem muss die zu testende Person mit auf dem Brustkorb verschränkten Armen so schnell wie möglich 5‑mal von einem Stuhl aufstehen und sich wieder hinsetzen. Insgesamt können in der SPPB max. 12 Punkte erzielt werden, wobei höhere Werte ein besseres Funktionsniveau anzeigen.

### Statistik

Die statistische Auswertung der Daten erfolgte anhand des Programms SPSS (IBM SPSS Statistics für Windows, Version 27.0. Armonk, NY, USA). Die Prüfung der Daten auf Normalverteilung wurde mit dem Kolmogorov-Smirnov-Test durchgeführt. Bei ordinalskalierten Daten sowie solchen, die die Voraussetzungen für den *t*-Test nicht erfüllten, erfolgte die Überprüfung der Unterschiedshypothese anhand des Mann-Whitney-U-Tests. Daten mit Nominalniveau wurden anhand eines Pearson-Chi-Quadrat-Test geprüft. Als signifikant wurden Unterschiede bei *p* < 0,05 bewertet. Als Maß der Effektstärke wurde bei Mann-Whitney-U-Tests der Pearson-Korrelationskoeffizient berechnet.

## Ergebnisse

### Beschreibung der Stichprobe

Auswertbare Datensätze lagen von *N* = 117 Personen vor. Das Alter der Gesamtstichprobe betrug im Durchschnitt *M* = 74,95 (*SD* = 6,74) Jahre. Der Frauenanteil lag bei 70,9 % (*n* = 83). Die Teilstichprobe „vor Pandemie“ umfasste *n* = 75 Personen, die Gruppe „während Pandemie“ bestand aus *n* = 42 Teilnehmenden. Keine dieser Personen war zum Zeitpunkt des Assessments akut mit dem SARS-CoV-2-Virus infiziert. Eine systematische Abfrage des Genesenenstatus erfolgte in unserer Klinik erst ab Juni 2020. Alle *n* = 17 Personen, die ab diesem Zeitpunkt eingeschlossen wurden, gaben an, in den letzten 6 Monaten vor Aufnahme keine nachgewiesene COVID-19-Infektion gehabt zu haben. Die Gruppen „vor“ bzw. „während der Pandemie“ unterschieden sich weder im Hinblick auf das Alter noch in Schmerzdauer oder Grad der Behinderung (Tab. 1).

**Table 1:** 

	Gruppe „vor Pandemie“	Gruppe „während Pandemie“	Unterschiedsprüfung
Alter *M (SD)*	75,17 (6,79)	74,55 (6,71)	*U* = 1514,50, *Z* = −0,344, *ns*
*Geschlecht, n (%)*			χ^2^(1) = 1,85, *ns*
Weiblich	50 (66,67)	33 (78,57)	–
Männlich	25 (33,33)	9 (21,43)	–
*Schmerzdauer, n (%)*			χ^2^(4) = 4,89, *ns*
1 Monat bis ½ Jahr	9 (12,00)	5 (11,99)	–
½ Jahr bis 1 Jahr	10 (13,33)	7 (16,67)	–
1–2 Jahre	8 (10,67)	5 (11,90)	–
2–5 Jahre	16 (21,33)	14 (33,33)	–
> 5 Jahre	31 (41,33)	9 (21,43)	–
Keine Angabe	1 (1,33)	2 (4,76)	–
*Grad der Behinderung (GdB), n (%)*			*U* = 1615,50, *Z* = −1,271, *ns*
90–100	2 (2,67)	1 (2,38)	–
70–80	12 (16,00)	6 (14,39)	–
50–60	17 (22,67)	4 (9,52)	–
30–40	7 (9,33)	3 (7,14)	–
Kein GdB	26 (34,67)	19 (45,24)	–
Keine Angabe	11 (14,67)	9 (21,43)	–

*ns* nicht-signifikant

### Charakteristika der Schmerzerkrankung

In beiden Gruppen wurden jeweils über 75 % der vergebenen Schmerzdiagnosen durch *Krankheiten des Muskel-Skelett-Systems und des Bindegewebes* ausgemacht. *Krankheiten des Nervensystems* lagen bei jeweils etwa 10 % der Personen in beiden Gruppen vor, weitere Diagnosekategorien wurden deutlich seltener vergeben (Tab. 2).

**Table 2:** 

		Gruppe „vor Pandemie“		Gruppe „während Pandemie“	
Diagnose (ICD-10)	Bezeichnung	*n*	%	*n*	%
A00–B99	Bestimmte infektiöse und parasitäre Krankheiten	0	0,00	3	3,49
G00–G99	Krankheiten des Nervensystems	20	11,43	9	10,47
H60–H95	Krankheiten des Auges und der Augenanhangsgebilde	0	0,00	1	1,16
M00–M99	Krankheiten des Muskel-Skelett-Systems und des Bindegewebes	135	77,14	65	75,58
N00–N99	Krankheiten des Urogenitalsystems	1	0,57	0	0,00
R00–R99	Symptome und abnorme klinische und Laborbefunde, die anderenorts nicht klassifiziert sind	11	6,29	2	2,33
S00–T98	Verletzungen, Vergiftungen und bestimmte andere Folgen äußerer Ursachen	4	2,29	0	0,00
–	Fehlerhafte Angabe im Entlassbrief	4	2,29	5	5,81

Es ergab sich kein signifikanter Unterschied zwischen den Teilstichproben in Bezug auf die Anzahl der ärztlich vergebenen Schmerzdiagnosen vor bzw. während der COVID-19-Pandemie (*M*_*vor*_ = 2,32 [*SD* = 1,36], *M*_*während*_ = 2,05 [*SD* = 1,01], *U* = 1452,00, *Z* = −0,729, *ns*).

**Figure 1:**
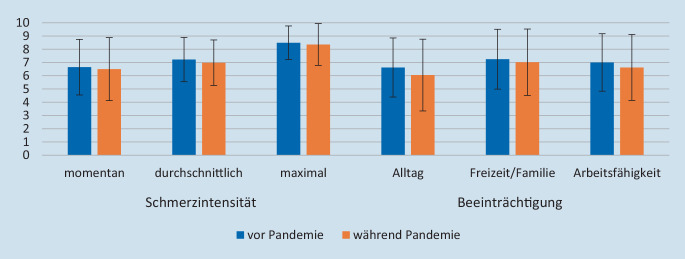

Beide Gruppen litten an moderaten bis starken Schmerzen mit entsprechender Beeinträchtigung (Abb. 1), wobei es keine signifikanten Unterschiede zwischen den Gruppen gab. Allerdings berichtete die präpandemische Teilstichprobe signifikant mehr Beeinträchtigungstage als die Gruppe, die während der Pandemie behandelt wurde (*M*_*vor*_ = 58,78 [*SD* = 34,95], *M*_*während*_ = 41,21 [*SD* = 33,63], *n* = 102, *U* = 852,00, *Z* = −2,50, *p* = 0,012*, *r* = −0,25).

### Psychische Beeinträchtigung

Bei 88 % der präpandemischen und 100 % der intrapandemischen Teilstichprobe wurde im Rahmen des interdisziplinären multimodalen Assessments die ICD-10-Diagnose F45.41 *Chronische Schmerzstörung mit somatischen und psychischen Faktoren* gestellt. In der präpandemischen Gruppe lag außerdem bei 4 % die Diagnose *Psychologische Faktoren oder Verhaltensfaktoren bei anderenorts klassifizierten Krankheiten* (F54) vor, bei 8 % konnte keinerlei psychische Erkrankung festgestellt werden.

Führend bei den nichtschmerzbezogenen psychischen Diagnosen waren die depressiven Störungen aus den Kategorien F32 *Depressive Episode* und F33 *Rezidivierende depressive Störung.* 40,0 % derer, die vor der Pandemie behandelt wurden, und 23,8 % jener, die während der Pandemie behandelt wurden, wiesen mindestens eine solche Diagnose auf. Bei einer Mehrzahl der behandelten Personen beider Teilstichproben (53,3 % bzw. 69,1 %) lag neben der schmerzbezogenen F‑Diagnose aber keine weitere psychische Störung vor. Zwischen den Teilstichproben ergab sich kein signifikanter Unterschied in Bezug auf die Gesamtzahl der vergebenen psychischen Diagnosen („F-Diagnosen“, *M*_*vor*_ = 1,56 [*SD* = 0,92], *M*_*während*_ = 1,38 [*SD* = 0,62], *U* = 1397,50, *Z* = −1,116, *ns*).

Im *Marburger Fragebogen für habituelles Wohlbefinden* (*MFHW*) ergab sich ebenfalls kein signifikanter Gruppenunterschied zwischen den präpandemisch behandelten Personen (*M*_vor_ = 10,03, *SD* = 8,25) und der während der Pandemie behandelten Gruppe (*M*_während_ = 10,70 [*SD* = 9,41], *n* = 114, *U* = 1469,00, *Z* = −0,16, *ns*). Im MFHW sind Werte von 10 oder niedriger als auffällig zu bewerten, was auf ein im Mittel ähnlich vermindertes Wohlbefinden in beiden Gruppen hindeutet.

**Figure 2:**
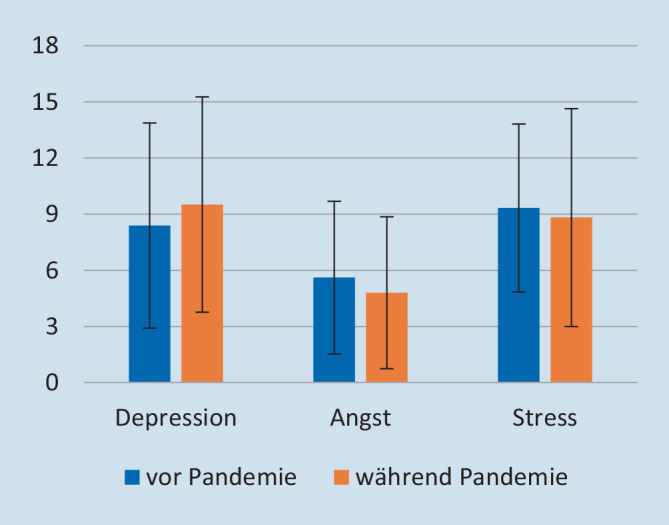

Die Ergebnisse der *DASS* sind in Abb. 2 dargestellt. Auch hier zeigten sich keine signifikanten Gruppenunterschiede. Die Cut-off-Werte von ≥ 10 für Depression und Stress sowie ≥ 6 für Angst werden im Mittel nicht überschritten.

### Körperliches Funktionsniveau

Die Teilstichprobe, die während der COVID-19-Pandemie behandelt wurde, erzielte zum Zeitpunkt des interdisziplinären multimodalen Assessments im Mittel bessere Werte in der *Short Physical Performance Battery (SPPB; M*_vor_ = 8,13, *SD* = 3,00; *M*_während_ = 9,57, *SD* = 2,85). Der Unterscheid erreicht das statistische Signifikanzniveau bei kleiner Effektstärke (*n* = 91, *U* = 672,00, *Z* = −2,54, *p* = 0,011*, *r* = −0,27).

## Diskussion

In dieser Arbeit konnten wir in einer Analyse von klinischen Routinedaten zum Zeitpunkt eines interdisziplinären multimodalen Assessments zeigen, dass sich die Gruppen von SeniorInnen vor und während der COVID-19-Pandemie wider Erwarten kaum in Schmerz, Beeinträchtigung und psychischem Befinden unterscheiden. Die präpandemische Gruppe wies sogar einen höheren Anteil an Depressionsdiagnosen auf, obwohl der Unterschied das statistische Signifikanzniveau nicht erreicht. Dazu passt, dass diese Gruppe im Vergleich zur intrapandemischen Gruppe im Mittel an signifikant mehr Tagen nicht dazu in der Lage war, ihren üblichen Aktivitäten nachzugehen. Auch das Funktionsniveau in der *Short Physical Performance Battery* war bei der intrapandemischen Gruppe eher besser als in der präpandemischen Gruppe. Beide Effekte sind allerdings als klein einzuordnen.

Basierend auf klinischen Erfahrungen und der Datenlage in der Literatur [8] hatten wir erwartet, dass SeniorInnen durch den Wegfall von sozialen Kontakten im familiären Umfeld und bei Freizeitaktivitäten und den Ausfall von körperlichen Betätigungsmöglichkeiten bzw. Physiotherapie in Schmerz und psychischem Wohlbefinden deutlich beeinträchtigter gewesen wären. Mehrere andere Untersuchungen berichten eine Verschlechterung von chronischen Schmerzen [11, 14] und stehen somit im Gegensatz zu unserem Befund. Allerdings sind die Daten aus anderen Ländern nur bedingt auf die Situation in Deutschland übertragbar, da sich der Schweregrad der Pandemie und die politischen Maßnahmen zum Infektionsschutz sowie allgemeine Strukturen in der Gesundheitsversorgung in verschieden Staaten deutlich unterscheiden. Auch unser multimodales Assessment und das daran anknüpfende Therapieangebot, auf das die PatientInnen bei Erhebung der Daten bereits Aussicht hatten, ist als länderspezifische Besonderheit zu sehen.

So lässt sich aus der vorliegenden Arbeit folgern, dass auch die vulnerable Gruppe von SeniorInnen mit chronischen Schmerzen, zumindest in einer Region im Norden von Bayern im Einzugsgebiet einer Universitätsklinik, nicht schlechter mit ihren Beschwerden umgehen konnte.

Menschen, die an chronischen Schmerzerkrankungen leiden, schneiden bei Erhebungen des Wohlbefindens und der psychiatrischen Komorbiditäten schlechter ab als gesunde Personen [7, 15]. Das allgemeine Wohlbefinden der hier untersuchten schmerzerkrankten Personen liegt deutlich unter dem von gesunden Personen (durchschnittlich 20 Punkte im *MFHW*), die *DASS*-Werte liegen knapp unter den Grenzwerten zur Auffälligkeit. Sie gehören somit zu einer vulnerablen Gruppe. Deren Outcome in der Pandemie ist variabel: Bu, Steptoe und Fancourt wiesen eine stärkere Belastung nach [3], während Hansen et al. keine vermehrte Beeinträchtigung durch die Maßnahmen zeigen konnten [10]. Eine mögliche Erklärung liegt laut den Autoren darin, dass sich der Lebensalltag dieser Menschen durch die pandemiebedingten Einschränkungen weniger veränderte als der von Personen, deren Alltag zuvor viele soziale Aktivitäten beinhaltet hatte.

Offensichtlich gibt es auch Gründe dafür, dass Menschen höheren Alters relativ gut mit der Pandemie umgehen können. SeniorInnen verfügen über eine deutlich größere Menge an Lebenserfahrung und sind dadurch in der Lage, über die Spanne eines bereits langen Lebens reflektieren zu können. Aktuelle Entwicklungen und Probleme können an vergangenen gemessen und in einen größeren Kontext eingeordnet werden. Die Studie von Bidzan-Bluma et al. [2] stellte außerdem fest, dass besonders ältere Menschen sich in Bezug auf die Pandemie optimistisch zeigten; eine Eigenschaft, die mit höherem Wohlbefinden in Zusammenhang steht [5]. Ein altersspezifischer Schutzfaktor könnte zudem die bereits erfolgte Berentung bzw. Pensionierung der untersuchten Personen sein, die ihre finanzielle Sicherheit gewährleistet [2].

### Stärken der Arbeit

Alle in dieser Arbeit ausgewerteten Daten wurden im Rahmen der regulären Patientenaufnahme und -behandlung erhoben; die Stichprobe unterliegt daher keiner Verzerrung hin zu einem überdurchschnittlich hohen Bildungsniveau oder der Verzerrung hin zu technisch versierteren älteren Personen, die in einigen Pandemiestudien zu sehen ist [2, 10].

Eine weitere Stärke der Arbeit liegt in der Verwendung des *DSF*, dessen Skalen vielfach validiert wurden [19], sowie in der Betrachtung verschiedener schmerzbezogener Aspekte aus unterschiedlichen diagnostischen Quellen.

Ein Großteil der bislang existierenden Studien zum Effekt der Pandemie beschränkt sich auf die ersten Monate nach ihrem Ausbruch; Arbeiten, deren Erhebungszeitraum eine längere Dauer der Pandemie umfasst, sind rar. Die umfassende Dauer der Datenerhebung ist daher als weitere Stärke der Arbeit anzusehen.

### Limitationen der Arbeit

Die größten Limitationen dieser Arbeit sind das retrospektive Design und die geringe und ungleiche Stichprobengröße, die dadurch bedingt ist, dass während der COVID-19-Pandemie aufgrund der geltenden Maßnahmen weniger Personen simultan behandelt werden konnten als davor. Dies hat eine eingeschränkte Reliabilität der Ergebnisse zur Folge. Das verwendete Between-Design erlaubt außerdem keinen Aufschluss über die intraindividuelle Entwicklung von Personen während der Pandemie. Auch lässt unsere Arbeit keine Schlüsse über die Effekte einer Erkrankung mit COVID-19 auf die untersuchten Parameter zu, da keine nachgewiesenen SARS-CoV-2-Infektionen in unserer Stichprobe bekannt waren.

Es ist zudem zu beachten, dass die Arbeit lediglich die Daten von in die Seniorengruppe aufgenommenen Personen umfasst. Personen, die aus Angst vor einer Infektion mit SARS-CoV‑2 keine ärztliche Hilfe aufsuchten, wurden im Rahmen dieser Arbeit nicht erfasst. Es existieren jedoch Hinweise darauf, dass besonders der Aufschub und der Ausfall von Schmerzbehandlungen einen negativen Effekt auf Schmerz und psychische Beeinträchtigung haben [12]. Aufgrund dieses möglichen Selektionseffekts sind die Ergebnisse dieser Arbeit eventuell positiver, als sie es für unbehandelte schmerzerkrankte Menschen höheren Alters wären. Dieser Effekt könnte möglicherweise noch dadurch verstärkt worden sein, dass die Aussicht auf die Teilnahme an einem strukturierten, mehrwöchigen Gruppenprogramm besonders für die intrapandemische Gruppe bereist psychisch entlastend gewirkt haben könnte.

## Fazit für die Praxis

- Entgegen unserer Erwartung zeigen unsere Daten keine Verschlechterung von Schmerz, psychischer Belastung und körperlichem Funktionsniveau von Menschen höheren Alters mit chronischen Schmerzstörungen während der COVID-19-Pandemie in einer universitären Tagesklinik.
- Unsere PatientInnen im höheren Lebensalter scheinen die coronabedingten Kontakt- und Ausgangsbeschränkungen gut kompensieren zu können, allerdings sind methodische Verzerrungen nicht auszuschließen. So beschränkt sich die vorliegende Arbeit auf Personen, die sich zur Behandlung an das Universitätsklinikum Würzburg begeben haben. Zur Validierung der Befunde sollten zukünftig auch Personen untersucht werden, die sich, eventuell auch aus Angst vor COVID-19, nicht aktiv zu einer Therapie gemeldet haben.
- Weitere, ggf. qualitative Untersuchungen sollten den Einfluss potenzieller Resilienzfaktoren, wie Zuversicht, Lebenserfahrung oder finanzieller Absicherung, untersuchen. Sollte sich dabei bestätigen, dass ältere Menschen über spezifische protektive Verarbeitungsmechanismen verfügen, ließen sich spannende Fragestellungen in Bezug auf die Trainierbarkeit dieser Faktoren in anderen Alters- oder besonders vulnerablen Gruppen entwickeln.
